# A molecular analysis of biclonal follicular lymphoma: further evidence for bone marrow origin and clonal selection

**DOI:** 10.1111/j.1600-0609.2009.01233.x

**Published:** 2009-05

**Authors:** Yuichi Nakamura, Yasutaka Sato, Katsuhiko Yoshida, Emi Kakegawa, Yoshihiro Ito, Atsushi Seyama, Hidekazu Kayano, Masami Bessho

**Affiliations:** 1 Department of Hematology, Saitama Medical UniversitySaitama, Japan; 2 Research Center for Genomic Medicine, Saitama Medical UniversitySaitama, Japan; 3 Department of Pathology, Saitama Medical UniversitySaitama, Japan

**Keywords:** follicular lymphoma, clonal selection, somatic hypermutation

## Abstract

We report a follicular lymphoma (FL) case presenting the coexistence of two tumor cell subpopulations in lymph node (LN) and bone marrow (BM), which exhibited an inverse pattern of immunoglobulin light (IgL) chain gene rearrangement and expression: Igκ−λ+ in LN and Igκ+λ− in BM. These tumor clones shared an identical *BCL2-IgH* recombination, accompanying t(14;18)(q32;q21) translocation, and an identical variable, diversity and joining segments joining with clone-specific *VH* somatic hypermutations on the untranslocated *IgH* allele. Our study provides further evidence that FL clones, originating from common progenitor cells, can be developed independently at different sites and with different IgL expression after immune selection.

Follicular lymphoma (FL) is the second most frequent type of non-Hodgkin’s lymphoma. The follicular growth pattern, cellular protein expression, such as CD10 and BCL6, and the accumulation of ongoing somatic hypermutation (SHM) within the immunoglobulin heavy (*IgH*) chain gene variable regions (*IgVH*) in FL indicate that the disease is a malignancy of germinal center B cells (1–3).

The characteristic with FL is the t(14;18)(q32;q21) chromosomal translocation, which juxtaposes the *BCL2* gene on chromosome 18 with the *IgH* on chromosome 14 and results in the dysregulation of the *BCL2* (4–6). The molecular anatomy around the t(14;18) breakpoints indicated that the translocation occurs as an error of the *D-JH* rearrangement at the pro-B cell stage of ontogeny (7, 8).

The inappropriate expression of BCL2 alone seems to be insufficient for malignant transformation of B cells and multistep process is required for FL development (2, 3). Several studies in FL cases based on idiotype expression and SHM analysis within the *IgVH* have indicated that t(14;18)-bearing B cells can migrate from bone marrow (BM) to germinal center of lymph node (LN) and be clonally expanded after antigenic selection (9–14). However, clonality analysis as to the immunoglobulin light (IgL) chain was reported in a limited case (15).

Here we report a FL case in which LN and BM tumor cells expressed different IgL chains. The results of molecular analysis indicated that the two cell subpopulations were derived from the common FL progenitor and developed into independent clones at different sites.

## Patient and methods

### Case report

A 51-yr-old woman was referred because of systemic lymphadenopathy. An inguinal LN biopsy was consistent with a FL, grade 2, according to the World Health Organization classification (1). Her BM biopsy sample presented the infiltration of FL.

The result of karyotypic analysis on LN cells was as follows: 51,XX,+2,add(7)(q32),+12,t(14;18)(q32;q21),+17,+21,+mar[3/4]/46,XX[1/4]. Interphase fluorescent *in situ* hybridization analysis of her BM cells revealed the juxtaposition of the *BCL2* with the *IgH* on 30% of cells analyzed.

### Flow cytometry

Cells obtained from LN and BM were analyzed by two-color immunofluorescence with a flow cytometer (FACSCalibur; Becton Dickinson, Mountain View, CA, USA) for expression of surface antigens. Analyses were done using monoclonal antibodies specific for CD3, CD5, CD10 and CD19 (Beckman Coulter, Miami, FD, USA); CD20 (Dako, Glostrup, Denmark); Igκ and λ chain (BD Biosciences, San Jose, CA, USA). Immunoreactivity was evaluated on CD45-gated cell populations.

### DNA isolation and Southern blotting

Genomic DNA was extracted from biopsied LN cells and BM mononuclear cells. DNA was also prepared from the patient’s peripheral blood mononuclear cells, in which no lymphoma cells were detected. An informed consent was obtained in accordance with the Declaration of Helsinki. After DNA digestion with *Hin*dIII (for the *IgH*), *Bam*HI (for the *Igκ*) and *Eco*RI plus *Hin*dIII (for the *Igλ*), Southern blotting was carried out by standard procedure. Probes were fluorescein-labeled using Gene Images Random Prime Labeling kit (GE Healthcare, Buckinghamshire, UK) and hybridization signals were detected using Gene Images CDP-star Detection kit (GE Healthcare). DNA probes for detection of the *IgH*, the *Igκ* and the *Igλ* gene rearrangement were as follows: JH, a 3.5-kb *Eco*RI-*Hin*dIII fragment; Cκ, a 2.5-kb *Eco*RI fragment; Cλ, a 0.7-kb *Bgl*II-*Eco*RI fragment containing the *Cλ3*.

### PCR and DNA sequence

The DNA was amplified by PCR using LA Taq Polymerase (Takara Bio, Otsu, Japan) for the BCL2/IgH rearrangement and the IgH gene analysis. The cycling conditions included an initial denaturation for 4 min at 94°C followed by 30 cycles of with 94°C denaturing for 30 s and 6 to 8 min annealing/primer extension at 68°C plus final extension 7 min at 72°C. Reaction mixture (50 μL) contained 100–200 ng DNA in LA-PCR reaction buffer with 0.4 μmol/L primers, 0.4 mmol/L dNTPs, 2.5 mmol/L MgCl_2_, and 2.5U LA Taq-Polymerase. The PCR products were directly cloned into pCR2.1-TOPO vector (Invitrogen, San Diego, CA, USA) and sequenced by an ABI Prism 310 Genetic Analyzer (Applied Biosystems, Foster City, CA, USA).

For detection of the *Igκ* deleting element (Kde) rearrangement, DNA was also amplified employing PCR system and primers previously reported by Stolz *et al.* (16).

### PCR primers for the BCL2/IgH rearrangement and the IgH gene analysis

For detection of the *BCL2*/*IgH* rearrangement, several previously reported sets of forward BCL2 and reverse IgH primers (17) were used for screening. For cloning of the chromosomal breakpoint, a new BCL2 forward primer, 5′-CAGATGAGCATGAATGGTACTGTACCG-3′, for 3′ region of the major breakpoint region (MBR) and IgH reverse primer (Sμ), 5′-ACATAAATGAGTCTCCTGCTCTTCATCAAG-3′, for switching μ region were employed. Additional reverse primers, 3′JH, 5′-CCCACAGGCAGTAGCAGAAAACAA-3′ and 5′JH, 5′-ATGGCAGAATGTCCATCCTCACCCCAC-3′ were used for further analysis.

**Figure 2:**
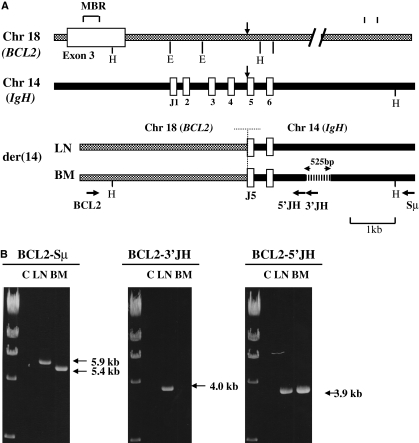
(A) Structure of the der(14) chromosome generated by t(14;18)(q32;q21) translocation. Vertical arrows show the chromosomal breakpoints. Deleted region in BM cells is shown by a dashed line. Open boxes are exons. Horizontal arrows indicate the locations of PCR primers. E, *Eco*RI; H, *Hin*dIII. (B) PCR detection of the *BCL2*-*IgH* fusion on der(14). DNA was amplified by primer sets as indicated. Arrows show the amplified fragments with size. Lanes C present DNA from the patient’s peripheral blood mononuclear cells.

For analysis of SHM, DNA was amplified using forward VH family-specific leader primers in conjunction with a reverse consensus JH primer as previously reported (18). A 5′ upstream primer within the *VH3-53* (5′VH3-53, 5′-GTCGTGTCTGATGAAGTCACACACTCAGAC-3′) was prepared on the basis of published sequence. For amplification of germline *VH3-53*, 3′VH3-53 primer (5′-CTGATAGGAGGAGACTCAGCAGG-3′), which annealed to the sequence 380 bp downstream from the 3′ heptamer nonamer recombination sequences, was also employed. The locations of primers used in this study were shown in Figs 2 and 3.

## Results

**Figure 1:**
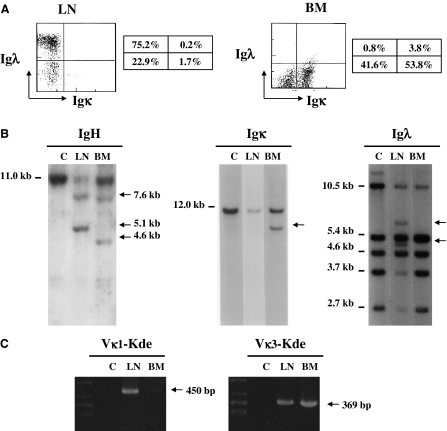
(A) Flow cytometric analysis of surface Igκ and Igλ chain expression on the patient’s LN and BM cells. (B) Southern blot analysis presenting *Ig* gene rearrangements. Lanes C present the germ line control. Arrows show the rearrange bands. (C) PCR detection of the Kde rearrangement. DNA was amplified by primer sets as indicated. Arrows show the amplified fragments with size. Lanes C present DNA from the patient’s peripheral blood mononuclear cells.

Flow cytometry analysis revealed that LN and BM tumor cells presented an inverse pattern in surface IgL chain expression; Igκ−λ+ in LN and Igκ+λ− in BM (Fig. 1A). The expressions of other surface antigens were similar in both samples (positive for CD10, CD19 and CD20, and negative for CD3 and CD5). Southern blotting presented monoclonal *Ig* rearrangements in both samples, but rearranged *IgL* gene was different between the two; the *Igλ* in LN and the *Igκ* in BM (Fig. 1B). In analysis of Kde configuration, *Vκ1-*Kde and *Vκ3-*Kde recombinations were detected in LN, while only *Vκ3-*Kde was detected in BM (Fig. 1C). Sequence around the *Vκ3-*Kde recombination junction was identical in both samples (data not shown).

On PCR screening using the previously reported primer sets (17), the chromosome 18 breakpoint was predicted to be located out of MBR in both LN and BM. A set of new BCL2 and IgH-Sμ primers amplified a 5.9-kb DNA fragment from LN and a 5.4-kb one from BM, respectively (Fig. 2). Sequence analysis revealed an identical *BCL2-JH5* recombination junction on both samples (data not shown), with the chromosome 18 breakpoint located at 5 kb downstream of the MBR (Fig. 2A). However, 525 bp deletion within *JH* region on chromosome 14 had occurred in BM cells, as ascertained by PCR using additional JH primers (Fig. 2B).

On Southern blotting, two rearrange bands were hybridized with JH probe in both LN and BM samples (Fig. 1B). Among these bands, the size of smaller bands (5.1 kb in LN and 4.6 kb in BM) coincided with the length of the cloned *Hin*dIII fragments containing the *BCL2-JH* junction (Fig. 2A). Thus, these bands were considered to be generated by t(14;18) translocation, with the difference in length due to the deletion in BM.

**Figure 3:**
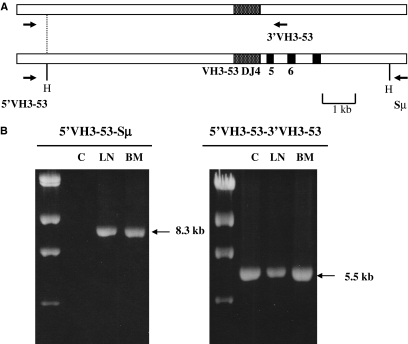
(A) Structure of the *VH3-53* region and the VDJ recombination on the untranslocated *IgH* allele. Horizontal arrows indicate the locations of PCR primers. H, *Hin*dIII. (B) PCR detection of the VDJ recombination and germ line *VH3-53* region. DNA was amplified by primer sets as indicated. Lanes C present DNA from the patient’s peripheral blood mononuclear cells as germ line control.

On the other hand, the comigrating 7.6 kb bands seemed to represent the variable, diversity and joining segments (VDJ) recombination on the untranslocated *IgH* allele. On PCR screening using VH leader primers (18), a set of VH3-specific primer and a JH primer amplified the rearranged fragment from both samples and the sequence of the 5′ part of amplified fragments showed the greatest homology with the *VH3-53* sequence. Then, DNA from tumor cells was subjected to amplification employing the 5′ VH3-53 forward primer and Sμ reverse primer (Fig. 3A). As shown in Fig. 3B, 8.3 kb bands were obtained from both samples by this primer set, which were ascertained as having *VH3-53* region by partial sequence analysis. The cloned PCR products contained the *Hin*dIII fragments corresponding to the bands on Southern blotting.

**Figure 4:**
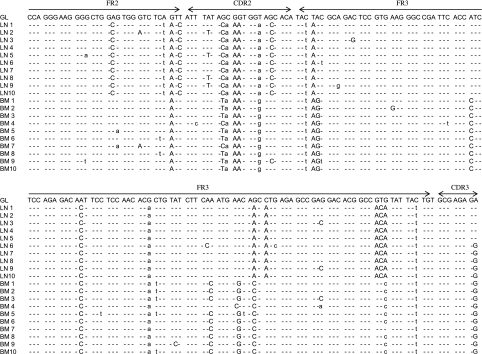
Nucleotide sequence comparison within the *VH3-53* region in LN and BM tumor cells. The nucleotide sequences obtained from bacterial isolates are compared with germline (GL) sequence from the patient’s peripheral blood mononuclear cells. Identity with GL is shown by dashes, replacement mutations are shown by uppercase letters and silent mutations are shown lowercase letters.

To characterize the SHM patterns in LN and BM, 10 sequences from independent bacterial isolates in each sample were analyzed in comparison with the germline. As shown in Fig. 4, base replacements were classified into three forms; common to both clones, LN or BM clone-specific and intraclonal sporadic ones. Germline sequence obtained from the patient’s peripheral blood mononuclear cells (analyzed on 10 bacterial isolates) was completely identical to the published one. When a clone-specific mutation was defined as one observed in more than 90% of bacterial isolates from a cell subpopulation, among the 293 nucleotides from FR1 to CDR3 region within the *VH3-53*, 12 (4.1%), 26 (8.9%) and 12 (4.1%) mutations were common to both, LN-specific and BM-specific, respectively.

## Discussion

Here we presented a FL case presenting the difference in IgL chain expression between the LN and BM tumor cells. The results of immunophenotypic and genetic studies coincided and confirmed that Igκ−λ+ cells in LN and Igκ+λ− cells in BM represented separate clones in the immunological viewpoint. These two tumor clones shared an identical *BCL2-IgH* recombination, accompanying t(14;18)(q32;q21) translocation, and an identical VDJ joining on the untranslocated *IgH* allele. In the *Igκ* gene analysis, these clones also shared an identical *Vκ3*-Kde recombination, but a fertile rearrangement involving *Cκ* was detected only in BM on Southern blotting and LN cells seemed to have biallelic abortive *Vκ*-Kde recombinations followed by the *Igλ* rearrangement. These observations indicated that the two clones were derived from the common progenitor cells at the stage before or during the *IgL* gene rearrangement in B-cell ontogeny, which harbored t(14;18) translocation.

Kobrin *et al.* (15) reported a FL case in which IgL expression of tumor cells changed from Igκ at diagnosis to Igλ at relapse and raised the possibility that Igλ-expressing cells would have arisen subsequent to a secondary IgL rearrangement that occurred in Igκ-expressing tumor cells, as SHMs were shared by both cell subpopulations. In our case, it is not likely that Igλ-expressing LN cells were converted from Igκ-expressing BM cells, as the concomitant *IgH* deletion on the der(14) chromosome in BM, which seemed to occur subsequent to the generation of the t(14;18) translocation, was not present in LN. On the other hand, results of the *Igκ* gene analysis including the Kde rearrangement indicated that Igκ-expressing BM cells would not be derived from Igλ-expressing LN cells. Thus, in this case, two tumor clones seemed to have developed independently, rather than that one had been converted from the other.

The SHM analysis within the *IgVH* revealed the occurrence of LN- and BM-specific base replacements. In the previous report on the SHM analysis in BM-infiltrated FL cases (19), it was shown that the majority of BM tumor cells were derivatives of ‘LN-inexperienced’ clone. Clone-specific SHMs in LN and BM in our case also indicate that these two clones were transformed by antigenic selection independently at the two sites.

However, some of base replacements within the *IgVH* observed in this case were common to the both clones and seemed to be tumor-related SHMs that generated at an early stage of development, as polymorphisms were denied by germline sequence from the patient’s non-lymphoma cells. As SHM is generally regarded as properties exhibited in mature B cells (20), the shared SHM patterns in this case would indicate that the common progenitor cells had reached at GC stage, and then developed into LN and BM tumor cells with independently occurring secondary IgL rearrangements (receptor revisions).

Alternatively, if pre-B cells had a potential of undertaking SHM as reported in cases with acute lymphoblastic leukemia L2 with t(14;18) (21), it would be probable that LN and BM tumor clones were originated from the common progenitor at the pre-B cell stage and developed with shared and clone-specific SHMs. It is unknown whether or not the occurrence of SHMs in early B cell stage was the feature with t(14;18)-bearing cells. Further investigation is needed to clarify this point.
